# Partnering With Patients to Improve a Multidisciplinary Sexual Dysfunction Program: A Survey of Patient Experience at a Single Institution

**DOI:** 10.7759/cureus.77087

**Published:** 2025-01-07

**Authors:** Noama Iftekhar, Patricia Mumby, Mary Lynn

**Affiliations:** 1 Department of Surgery, University of Nevada, Las Vegas School of Medicine, Las Vegas, USA; 2 Department of Behavioral Health, Loyola University Medical Center, Maywood, USA; 3 Department of Obstetrics and Gynecology, Loyola University Medical Center, Maywood, USA

**Keywords:** female sexual health, multidisciplinary, multidisciplinary decision-making, sexual dysfunction, sexual health

## Abstract

Background

Sexual dysfunction is highly prevalent. The Loyola University Medical Center Sexual Wellness Program (LU-SWP), a multidisciplinary program, uses a biopsychosocial and educational model to collaborate with patients in their treatment.

Objective

This study aims to examine the reported satisfaction scores for the LU-SWP and its activities based on a patient survey completed at the end of the program.

Patient involvement

Patients of the LU-SWP completed surveys gauging their opinions on presentations given and techniques used during the 6-week program. These surveys aided in program adjustments that increased patient-centeredness.

Methods

It is a retrospective assessment of patient satisfaction scores of the LU-SWP from 2014 to 2020.

Results

Eighty-five of the 90 participants completed the survey, giving a 94% response rate. The mean age of the participants in this program was 49.8 years (SD = 13.768). The mean program satisfaction score was 8.51 (SD = ±1.43).

Conclusions

Because of this study, patient satisfaction scores aided in updating aspects of the LU-SWP. The scores give patients a voice in their care and allow previous patients to act as advocates for future patients.

## Introduction

Sexual dysfunction is highly prevalent in both men and women. Worldwide, it affects 41% of adult women and up to a third of adult men [1-4]. A global survey found that the majority of men (80%) and women (60%) report that sex is an important aspect of quality of life [5]. Factors contributing to sexual dysfunction can vary [6-10]. Given the diverse diagnoses involved, a biopsychosocial approach best addresses the range of factors associated with sexual dysfunction [11-13].

Currently, several studies examine prostate cancer survivors’ satisfaction scores as part of quality improvement programs for sexual health [14-15]. By soliciting patient feedback, healthcare providers can more accurately address potential concerns with their treatment and effectively collaborate with their patients [15-18].

This study adds to the literature on the multidisciplinary treatment approach for sexual dysfunction, using a single clinic and the collaboration of several specialties to treat couples experiencing sexual dysfunction. Implementing satisfaction scores allows for the modeling of a practice that is patient-centered, especially in a field as sensitive and personal as sexual dysfunction.

## Materials and methods

This project was approved by the Institutional Review Board. The Loyola University Medical Center Sexual Wellness Program (LU-SWP) is a multidisciplinary program that unites psychologists, gynecologists, urologists, dieticians, physical therapists, yoga instructors, and advanced practice nurses in the diagnosis and treatment of sexual dysfunction using a biopsychosocial approach [13]. The program runs for six consecutive weeks. Patients and their partners present weekly to meet with their therapist dyad (an attending or senior sex therapist and a fellow or resident/intern). Couples receive weekly assignments to encourage the transfer of knowledge and skills gained in sessions to home life, including a “sexy surprise” (i.e., specific romantic and/or sensual gestures) for each other and sensate focus activities. The therapy dyad tailors treatment approaches and in-session/home exercises to the couple’s needs. Adult patients in a heterosexual relationship are referred by their primary care physician, obstetrician, psychiatrist, or urologist to our clinic. No patients are excluded from treatment.

A total of 90 adult patients, amounting to 45 heterosexual adult couples, were included in the study. Of these, five patients elected to opt out of study participation. Patients of the LU-SWP completed surveys gauging their opinions on educational presentations and sex therapy techniques used during the 6-week program. The surveys aided in program adjustments that increased patient-centeredness. At the completion of the program, patients completed an investigator-developed tool with twelve items to assess satisfaction with the program overall and with specific components. These aspects were ranked from 1 to 5, with 1 being least helpful and 5 being very helpful. Participants also completed a section asking them to rate the program on a scale from 1 to 10, with 1 being least satisfied and 10 being most satisfied with the program. The surveys are anonymous.

LU-SWP patient satisfaction scores were collected at the end of the six-week program from 2014 to 2020.

The satisfaction scores were then recorded in RedCap. From RedCap, data was exported to Excel for data analysis purposes. We computed quantitative statistics including mean, range, and standard deviation for the age of our participants. We also calculated the percent for most and least helpful activity for purposes of comparison.

## Results

**Figure 1:**
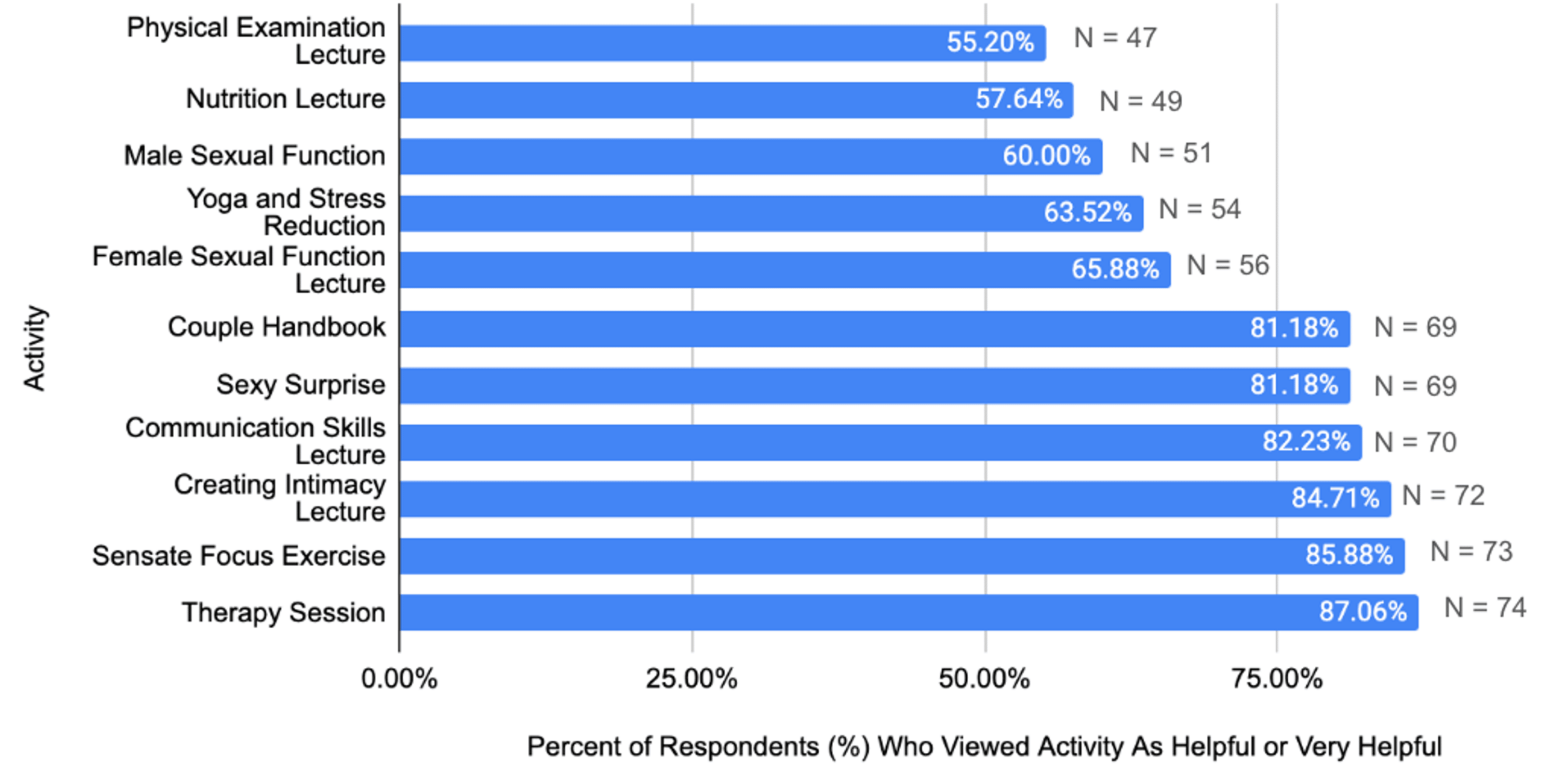
Percentage (%) of respondents (n=85) who viewed each activity as Helpful or Very Helpful. Our figure displays the percentage of respondents within each bar. Above the bar, (N = #) indicates the number of respondents who viewed the activity as helpful or very helpful.

**Figure 2:**
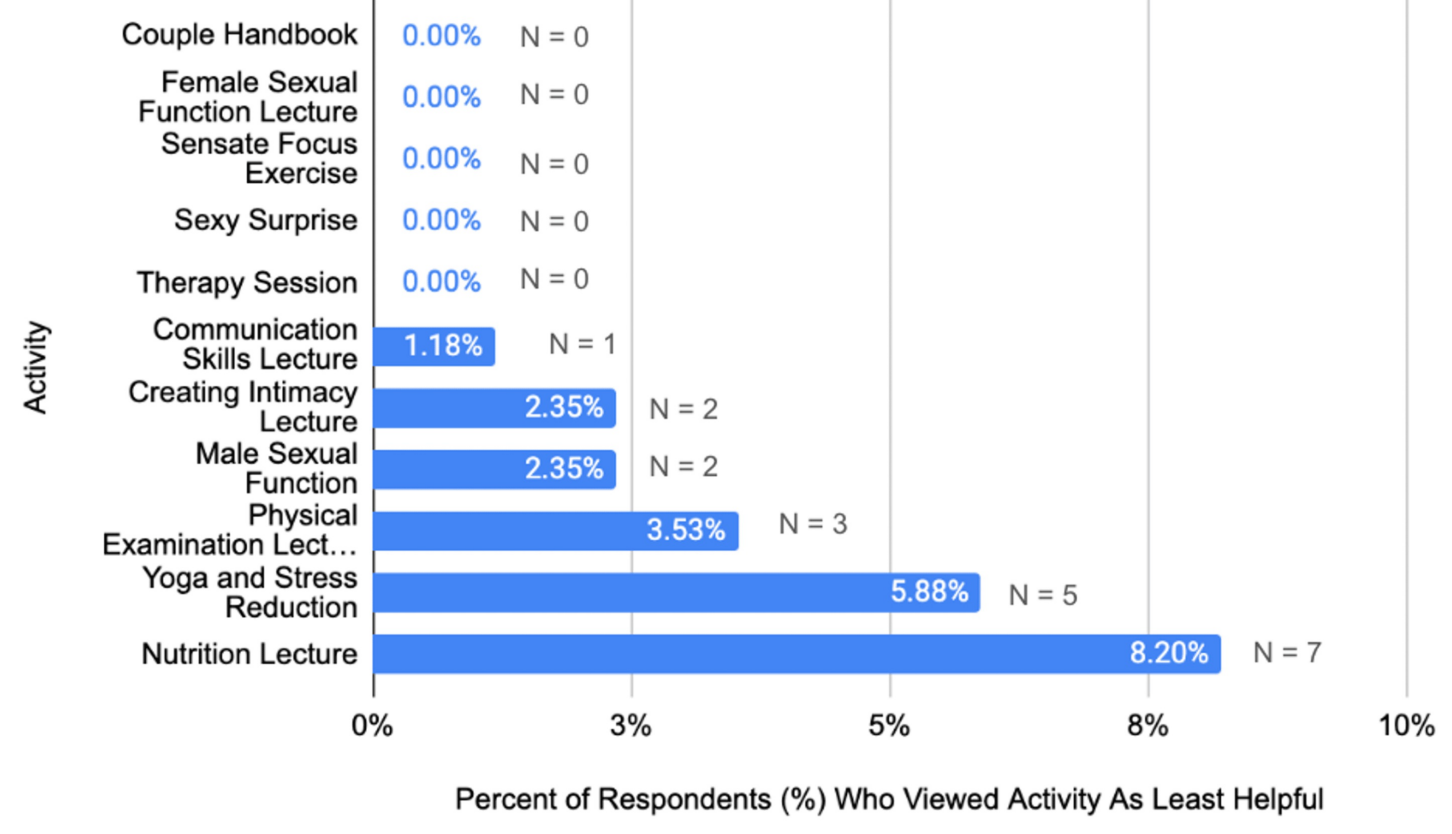
Percentage (%) of respondents (n = 85) who viewed each activity as Least Helpful. 0 out of 85 patients rated the Couple Handbook, Female Sexual Function Lecture, Sensate Focus Exercise, Sexy Surprise, or Therapy Session as the least helpful activity on a scale of 1 to 5. Our figure displays the percentage of respondents within each bar. Above the bar (N = #) indicates the number of respondents who viewed the activity as helpful or very helpful.

Of the 90 participants in the program, 85 surveys were returned (94% response rate). All of these responses were included in the analysis. Table 1 displays the demographic characteristics of the participants. Figure 1 presents participants' satisfaction scores for activities rated as helpful or very helpful. Figure 2 contrasts the percentage of participants who viewed activities within the LU-SWP as least helpful. The final section of our survey asked respondents to rank activities in the program on a scale of 1 to 5 (1 being not helpful, 5 being very helpful). All activities currently featured in the LU-SWP six-week program received a positive response, with 50% or more of the patients ranking them as helpful or very helpful.

**Table 1:** Demographics of survey respondents.

Characteristics		Value (N = Number)	Percent (%)
Age (years)		Mean = 49.8 years (SD 13.768) Range 25-77	
Gender (n = number, %)	Men	42	49.4
	Women	43	50.6
Ethnicity (n = number, %)	Caucasian	65	76.4
	African American	8	9.4
	Hispanic	7	8.2
	Asian	3	3.5
	Other	2	2.5

The satisfaction survey used by our team was a four-part paper survey. The initial question used a scale from 1 to 10, with 1 being 'worst satisfaction' and 10 as 'highest level of satisfaction.' Overall, the patient satisfaction scores showed a favorable response to the multidisciplinary psychoeducational treatment. Among participants, 79 ranked the program at a '5' or greater on the scale, and overall were highly satisfied with the program, with a mean satisfaction score of 8.51 (SD ± 1.43).

The therapy sessions were ranked as the most helpful component of the program, with 87% ranking the sessions as helpful or very helpful. The sexual function lectures were found helpful or very helpful by most respondents - 60% for the Male Sexual Function Lecture and nearly 66% for the Female Sexual Function Lecture. Sixty-three percent of respondents reported that the Yoga and Stress Reduction series was helpful or very helpful. Less favorable responses were recorded for the physical examination (55.2%) and nutrition lecture (57.64%).

Our findings suggest that sensate focus, a longstanding and robust technique to enhance intimacy, was a crucial component of the sex therapy, with 85.88% viewing the activity as helpful or very helpful.

## Discussion

Sexual dysfunction encompasses a variety of conditions: anorgasmia, pain during intercourse, lack of arousal, and hypoactive sexual desire or lack of sexual interest [17]. Sexual disorders are influenced by a multitude of internal and external factors, including medical and psychiatric comorbidities. Given the high reported prevalence of sexual dysfunction in the literature and the high levels of unreported dysfunction, sexual dysfunction is ultimately a public health concern.

The current study helps to address the limited literature on patient satisfaction with sexual wellness programs. Patients’ perspectives and the level at which they engage in the program are important aspects of the couple’s overall treatment. 

A recent pilot study conducted at the San Diego Sexual Medicine and Mayo Clinic Women’s Health Clinic evaluated female patient-reported satisfaction from a multidisciplinary sexual dysfunction treatment team [19]. Eighty-two percent of respondents to an emailed survey reported moderate and/or great benefit from a multidisciplinary team; 84.1% endorsed that they were somewhat and/or very satisfied with their treatment.

Similar to the San Diego/Mayo study, our team used a survey to determine patient satisfaction at the conclusion of the treatment program. Most patients (>80%) rated multiple aspects of the program as helpful or very helpful, reflecting the satisfaction scores achieved in the San Diego/Mayo study.

However, unlike the San Diego/Mayo study, our participant cohort included both males and females. Additionally, while the San Diego/Mayo study only included participants who required both a physical and sexual therapist, our study included all enrolled participants. Surveys were administered on the final day after treatment and were anonymous. Researchers in the San Diego/Mayo study emailed surveys after the completion of the study. Furthermore, the LU-SWP is a single-center program, which, as a result, includes a smaller sample size of participants.

The most helpful activity was the therapy session, with 87% viewing it as helpful or very helpful. Previous studies also endorsed how one-on-one therapy sessions are ranked highly in multidisciplinary programs. A thorough biopsychosocial interview of the partners, physical examination, and self-report questionnaires have been recommended for determining the root of sexual difficulties [19]. Cognitive-behavioral therapy (CBT) has shown tremendous benefit in treating sexual dysfunction, especially in women with pelvic pain disorders. CBT has demonstrated improved penetrative sexual intercourse in women with vestibulodynia and lifelong vaginismus [19-20]. All of these assessments were completed during the initial two therapy sessions of the LUC-SWP program. Additionally, a multidisciplinary study found that the amount of sensate focus during the last week of treatment was the strongest predictor of successful behavioral treatment for sexual dysfunction [21-26].

The patient satisfaction scores have aided in updating aspects of the LU-SWP. Based on our program surveys, we removed two lecture series, including yoga and nutrition. The scores give patients a voice in their care and allow previous patients to act as advocates for future patients. French hospitals have required patient satisfaction evaluations for the past two decades, and German hospitals have similarly mandated this evaluation since 2005 [27-28]. Recently, patient feedback has become a significant measure for quality improvement, but globally there is still an inadequate use of patient surveys [24, 25-26]. Patient satisfaction affects healthcare outcomes. Patients who are satisfied with their health services tend to comply more actively with treatment and take more initiative regarding their own health [26]. Questionnaires traditionally include both qualitative and quantitative components. These surveys not only function as an evaluation of a patient's perceptions of their treatment but also provide context to the care received. Their responses create an outlet for shared decision-making. Furthermore, satisfaction surveys allow for the evaluation of newly implemented programs in patient care [27-28].

Limitations of our study include the lack of generalizability due to the homogeneous population (primarily Caucasian) and the fact that this study was conducted at a single center. Our study is retrospective, and because the data was collected in the past, it can be difficult to establish a cause-and-effect relationship. Our study was relatively small, with only 85 patients, which can make it challenging to truly extrapolate and determine a concatenation of events. Our study lacked a control group for comparison. Furthermore, because of the anonymous nature of these surveys, differences between race and satisfaction cannot be accounted for.

Similarly to the San Diego/Mayo Clinic study, our current report does not discuss how the changes made to the program have ultimately altered satisfaction scores.

## Conclusions

When treating sexual dysfunction, a multidisciplinary approach is recommended over the traditional model of individual providers, since it addresses the complex, multidimensional nature of the issue. A couple-oriented approach is also preferred to an individual one. In addition to these approaches, the biopsychosocial model, which considers the patient as a whole and incorporates biological factors, cultural values, emotional needs, social relationships, medication history, and overall wellness, is utilized. The LU-SWP addresses this complex interrelationship through didactics, couple's sex therapy, and 'home assignments' led by an interdisciplinary team of mental health providers, gynecologists, and urologists.

## References

[1] McCool ME, Zuelke A, Theurich MA, Knuettel H, Ricci C, Apfelbacher C (2016). Prevalence of female sexual dysfunction among premenopausal women: a systematic review and meta-analysis of observational studies. Sex Med Rev.

[2] McCool-Myers M, Theurich M, Zuelke A, Knuettel H, Apfelbacher C (2018). Predictors of female sexual dysfunction: a systematic review and qualitative analysis through gender inequality paradigms. BMC Womens Health.

[3] Rosen RC (2000). Prevalence and risk factors of sexual dysfunction in men and women. Curr Psychiatry Rep.

[4] Lewis RW, Fugl-Meyer KS, Bosch R, Fugl-Meyer AR, Laumann EO, Lizza E, Martin-Morales A (2004). Epidemiology/risk factors of sexual dysfunction. J Sex Med.

[5] Mulhall J, King R, Glina S, Hvidsten K (2008). Importance of and satisfaction with sex among men and women worldwide: results of the global better sex survey. J Sex Med.

[6] Parmet S, Lynm C, Glass RM (2004). JAMA patient page. Male sexual dysfunction. JAMA.

[7] Dai H, Wang J, Zhao Q (2020). Erectile dysfunction and associated risk factors in male patients with ischemic stroke: a cross-sectional study. Medicine (Baltimore).

[8] Maiorino MI, Bellastella G, Esposito K (2014). Diabetes and sexual dysfunction: current perspectives. Diabetes Metab Syndr Obes.

[9] Lev-Sagie A, Witkin SS (2016). Recent advances in understanding provoked vestibulodynia. F1000Res.

[10] Krakowsky Y, Grober ED (2018). A practical guide to female sexual dysfunction: An evidence-based review for physicians in Canada. Can Urol Assoc J.

[11] Bitzer J, Platano G, Tschudin S, Alder J (2007). Sexual counseling for women in the context of physical diseases: a teaching model for physicians. J Sex Med.

[12] Basson R, Wierman ME, van Lankveld J, Brotto L (2010). Summary of the recommendations on sexual dysfunctions in women. J Sex Med.

[13] Lynn M, Iftekhar N, Adams W, Mumby P (2023). Multidisciplinary approach to the treatment of sexual dysfunction in couples using a biopsychosocial model. J Sex Med.

[14] Matthew AG, Trachtenberg LJ, Yang ZG (2022). An online Sexual Health and Rehabilitation eClinic (TrueNTH SHAReClinic) for prostate cancer patients: a feasibility study. Support Care Cancer.

[15] Wittmann D, Varlamos C, Rodriguez-Galano N (2022). Developing a patient-centered model of prostate cancer care: patient satisfaction with a survivorship program embedded in urologic-oncologic care. Urology.

[16] Greco M, Brownlea A, McGovern J (2001). Impact of patient feedback on the interpersonal skills of general practice registrars: results of a longitudinal study. Med Educ.

[17] Shaeer O, Skakke D, Giraldi A, Shaeer E, Shaeer K (2020). Female orgasm and overall sexual function and habits: a descriptive study of a cohort of U.S. women. J Sex Med.

[18] Rullo J, Faubion SS, Hartzell R (2018). Biopsychosocial management of female sexual dysfunction: a pilot study of patient perceptions from 2 multi-disciplinary clinics. Sex Med.

[19] Brotto LA, Bitzer J, Laan E, Leiblum S, Luria M (2010). Women's sexual desire and arousal disorders. J Sex Med.

[20] Brotto LA, Bergeron S, Zdaniuk B (2019). A comparison of mindfulness-based cognitive therapy vs cognitive behavioral therapy for the treatment of provoked vestibulodynia in a hospital clinic setting. J Sex Med.

[21] van Lankveld JJ, Granot M, Weijmar Schultz WC (2010). Women's sexual pain disorders. J Sex Med.

[22] Linschoten M, Weiner L, Avery-Clark C (2016). Sensate focus: a critical literature review. Sex Relat Therap.

[23] Sarwer DB, Durlak JA (1997). A field trial of the effectiveness of behavioral treatment for sexual dysfunctions. J Sex Marital Ther.

[24] Ramage M (1998). ABC of sexual problems: management of sexual problems. BMJ.

[25] Al-Abri R, Al-Balushi A (2014). Patient satisfaction survey as a tool towards quality improvement. Oman Med J.

[26] Byers ES, MacNeil S (2006). Further validation of the interpersonal exchange model of sexual satisfaction. J Sex Marital Ther.

[27] Schöpf AC, Vach W, Jakob M, Saxer F (2019). Routine patient surveys: patients' preferences and information gained by healthcare providers. PLoS One.

[28] Sack C, Scherag A, Lütkes P, Günther W, Jöckel KH, Holtmann G (2011). Is there an association between hospital accreditation and patient satisfaction with hospital care? A survey of 37,000 patients treated by 73 hospitals. Int J Qual Health Care.

